# How Have Quality Improvement Strategies Been Adopted and How Has Impact Been Assessed in Care Homes for Older People? A Systematic Search and Narrative Review

**DOI:** 10.1111/opn.70036

**Published:** 2025-09-05

**Authors:** Fiona Cowdell, Hannah Harvey, Judith Dyson

**Affiliations:** ^1^ Faculty of Health, Education and Life Sciences Birmingham City University Birmingham UK

**Keywords:** care homes, implementation, knowledge mobilisation, older adults, quality improvement, systematic review

## Abstract

**Introduction:**

We conducted a systematic search and narrative review of quality improvement in care homes. Our aim was to examine how quality improvement strategies have been adopted and how impact has been assessed in care homes for older people.

**Methods:**

Following PRISMA guidelines, we conducted systematic searches of the electronic databases Cumulative Index to Nursing and Allied Health Literature (CINAHL), Medline, PsycINFO and Applied Social Sciences Index & Abstracts (ASSIA) (2019–2024). We replicated the search strategy of a previously published review. Three co‐authors undertook selection and data extraction.

**Results:**

Forty‐four articles were included describing varied stages of quality improvement initiatives in care homes. The United States produced the largest number of studies. Quality improvement strategies were often poorly reported. Included papers reported stages of quality improvement from inception to evaluation. Most aimed for improved clinical outcomes, mainly those subject to external scrutiny. Few studies reported impact robustly. Quantitative measures, surveys and qualitative data were reported alongside staff‐reported changes. There was no evidence of sustained improvement. There is some evidence of the use of theories, models and frameworks usually associated with implementation and knowledge mobilisation.

**Conclusion:**

Current improvement practice is inconsistent and having limited impact.

**Implications for Practice:**

We recommend any initiative to enhance resident experience and outcomes should involve establishing a project team, moving away from a deficit model, prioritising areas for improvement, identifying best practice, deciding how to measure improvement, understanding the challenges to best practice, co‐designing strategies to effect change, sustaining the improvement, sharing learning and providing clear, detailed and accessible reporting.

**Summary**

- What does this research add to existing knowledge in gerontology?
  ○ Many quality improvement (QI) initiatives in care homes have modest, short‐lived impacts, with poor reporting that limits replication.
  ○ QI efforts often focus on clinical outcomes rather than residents' preferences. There is a lack of resident involvement in prioritising QI strategies, emphasising the need for a more person‐centred approach.
  ○ The review offers insight into the application of various theoretical frameworks (e.g., PDSA cycles, COM‐B, knowledge mobilisation), expanding the understanding of how different strategies can support quality care in gerontology.
  ○ The research promotes the involvement of frontline staff and residents in designing QI strategies tailored to the specific context of care homes, offering practical recommendations for more sustainable improvements.
- What are the implications of this new knowledge for nursing care for and with older adults?
  ○ There is a need for increased staff engagement and ownership of QI initiatives to improve care outcomes, rather than relying on external facilitation.
  ○ It is important to move beyond clinical outcomes to include resident wellbeing and preferences as central goals in quality improvement efforts.
  ○ Co‐designing care strategies with both staff and residents could help to ensure that care meets the actual needs and preferences of older people.
- How could the findings be used to influence practice, education, research, and policy?
  ○ Regulatory frameworks could revise care home quality standards promote long‐term sustainability of QI efforts and incorporate resident‐centred measures to ensure that QI initiatives align with what matters most to them.
  ○ Care homes may benefit from engaging frontline staff and residents in identifying and sustaining improvements in care.
  ○ Future research could explore long‐term impacts of QI on resident experience. Educational programmes could be developed for care home staff to support them to understand and implement evidence‐based practices.

## 1 Introduction

Care homes provide accommodation and personal care, with or without nursing, most often to older people who are no longer able to manage in their own homes (Social Care Institute for Excellence 2024). Compared to larger healthcare settings, such as hospitals, care homes are generally smaller, less well‐resourced, and have higher proportions of staff providing care that are not required to be registrants of a professional body or hold specific care qualifications. In the United Kingdom, there are approximately 11,737 care homes with almost half a million residents aged 65 and over, and this is projected to increase with the ageing population (Office for National Statistics 2021).

Internationally, there is variation in the way care homes are funded, deliver care and how quality is measured. For example, in England, care homes are assessed for the quality of care they provide by the Care Quality Commission (CQC), the independent regulator of health and social care. Standards are judged on metrics encompassing safety, effectiveness, dignity, responsiveness and leadership (CQC 2024b). Care homes are judged as outstanding, good, requires improvement or inadequate. In July 2024, 19.3% of all care homes with ratings either scored ‘requires improvement’ or ‘inadequate’ (CQC 2024a). For decades, reports have shown residents' experiences are variable, and they sometimes experience poor access to a range of other health services (Smith et al. 2015).

Care homes are the resident's *home*. All care should be provided with dignity and according to resident preference and wellbeing. There is evidence, or best practice guidance, to support provision of many aspects of care, for example, skin care, nutrition, hydration, falls prevention and post‐falls assessment. Provision of high‐quality care is in part dependent on the ability to deliver care in accordance with existing evidence and guidance. A range of approaches can support improvements in care quality, including quality improvement (QI), implementation and knowledge mobilisation. QI is commonly used to optimise care delivery and is defined as a ‘systematic and coordinated approach to solving a problem using specific methods and tools with the aim of bringing about a measurable improvement’ (Jones et al. 2021). It emphasises empowering frontline staff through iterative change (typically using Plan, Do, Study, Act (PDSA) cycles) and continuous testing and measurement (often with statistical process control measures) (Institute for Healthcare n.d.). Implementation focuses on the process of integrating evidence‐based practices into routine care, ensuring that interventions are adopted and sustained in real‐world settings (Eccles and Grimshaw 2004). Knowledge mobilisation, on the other hand, refers to the movement of research and best practices across different settings to catalyse change and improve care delivery (Wye et al. 2019). These are widely used umbrella terms each encompassing a range of evidence to practice strategies. While QI is well‐documented in secondary care, its application in care homes is less understood and the role of implementation and knowledge mobilisation in this context remains underexplored. Contextual factors, such as setting and staffing, influence the way interventions designed to improve practice are adopted (Dixon‐Woods et al. 2012).

A recent scoping review of QI in care homes from 2000 to 2019 included 75 papers to identify participating occupational groups, resident‐level interventions and evaluation measures and outcomes (Chadborn et al. 2021). The authors report a steady increase in publications over each 5‐year period. Most papers emanated from the United States and described or evaluated a single QI project. The challenge of comprehensive reporting of QI initiatives was noted by the authors, indeed 15 articles came from six studies which used multiple publications to present their work. The authors conclude there is no obvious relationship between occupational group, interventions, evaluation measures and outcomes. Reporting was of limited quality in included papers. These authors recommend (i) a more robust approach to reporting QI initiatives, using tools such as CONSORT (Antes 2010) and SQUIRE 2.0 checklists (Ogrinc et al. 2016) (which were developed after some of the included papers were published), (ii) better reporting of outcomes and (iii) more focus on development of QI skills in care home staff as opposed to external facilitation (Chadborn et al. 2021). Our narrative review builds directly on this scoping review with the aim of examining how QI strategies have been adopted and how impact has been assessed in care homes for older people. From our findings we offer explicit recommendations to enhance resident experience and outcomes.

## 2 Materials and Methods

### 2.1 Search Strategy and Selection Criteria

This review was conducted in accordance with the Preferred Reporting Items for Systematic Review and Meta‐analyses (PRISMA) statement (Page et al. 2021) (Data S1). The search strategy was informed by Chadborn et al. (2021) and adapted in accordance with this being a narrative rather than a scoping review. The electronic databases Cumulative Index to Nursing and Allied Health Literature (CINAHL), Medline, PsycINFO and Applied Social Sciences Index & Abstracts (ASSIA) were searched to capture quality improvement projects in care home settings with residents aged over 65 years using the following terms:

“quality improvement” OR “total quality management” OR “PDSA” OR “process improvement” OR “health services research” OR “quality indicators, health care” OR “model for improvement” OR “six sigma”

**AND**

“long‐term Care” OR “LTCF” OR “institutional care” OR “skilled nursing facilities” OR “SNF” OR “residential home” OR “care home” OR “institutionalization” OR “residential facilities” OR “homes for the aged” OR “nursing homes”

A subject specialist research librarian was consulted to support search construction. A protocol was not prepared and the review was not registered. The search was conducted in August 2024 and, to avoid duplication of the review conducted by Chadborn et al. (2021), only included publications after January 2019. The decision was made to exclude grey literature to maintain methodological consistency and avoid potential biases from unpublished or non‐peer‐reviewed sources. The review was limited to items available in the English language. Full search details are in Data S2.

### 2.2 Selection Process

Study titles and abstracts were independently screened against the eligibility criteria (Table 1), with reasons for exclusion documented for each record. At both the title and abstract review stages, each author screened a third of the papers individually. Additionally, to ensure consistency in decision‐making, all three authors independently reviewed the same set of 50 papers and discussed their assessments. Discrepancies were minimal and were resolved through discussion until a consensus was reached. Full texts were obtained for papers that were not excluded at this stage, and the screening process was repeated.

**TABLE 1:** Review eligibility criteria.

Inclusion criteria	Exclusion criteria
Studies reporting quality improvement Conducted in care homes with or without nursing Peer‐reviewed Empirical studies Published from January 2019 onwards English language	Secondary analyses Grey literature Published pre‐2019 Not available in English language

### 2.3 Analysis

Data were extracted by all three authors using a bespoke data extraction sheet in which we recorded: aim, approach, rationale and theory, any improvement strategies and evaluation or impact (Data S3). We conducted a narrative review of included papers according to the focus of our review following the principles of providing a rationale, clarifying boundaries scope and definition, explaining analysis and interpretation and discussing strengths and limitation of the review (Sukhera 2022).

## 3 Results

**FIGURE 1:**
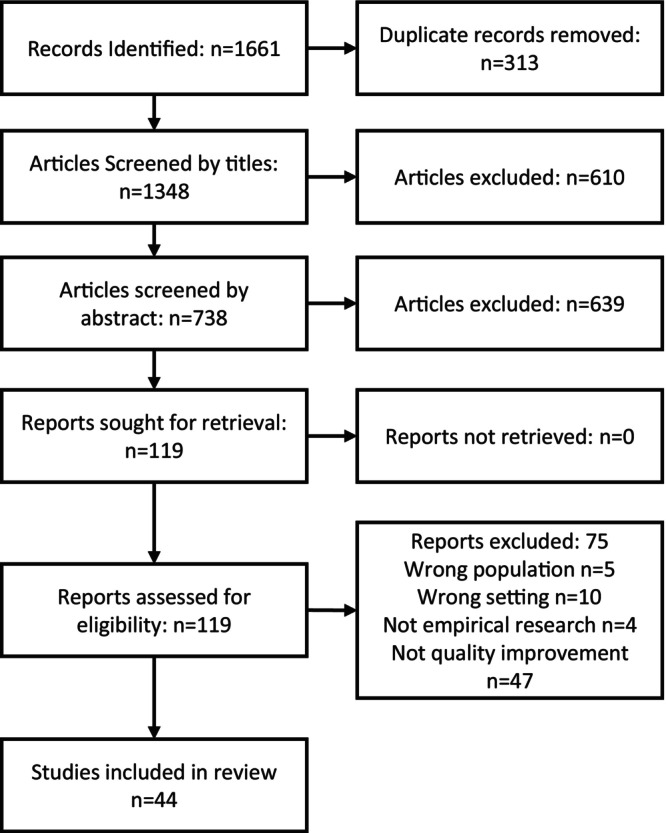
Prisma flow diagram.

The search returned a total of 1661 articles; 313 of these were duplicates, leaving 1348 to be screened. Forty‐four met the inclusion criteria (Figure 1).

### 3.1 Characteristics of Included Papers

Table 2 offers a summary of included studies. Most studies were conducted in the United States (*n* = 19) (Abbott et al. 2022; Au et al. 2019; Baluyot et al. 2022; Berning et al. 2021; Calderon et al. 2021; Douglas and Brush 2022; Harbison and Mwesige 2019; Hickman et al. 2020; Jackson et al. 2019; Jenko et al. 2019; Joy et al. 2021; Kay et al. 2023; Maguire et al. 2021; Mills et al. 2019; Ouslander et al. 2021; Pimentel et al. 2020; Porter et al. 2021; Toles et al. 2021; Volk et al. 2020). Other countries included United Kingdom (*n* = 7) (Anaba‐Wright and Kefas 2020; Collins et al. 2019; Damery et al. 2021; Hockley et al. 2019; Lavallée et al. 2019a, 2019b; Wilson et al. 2019), Canada (*n* = 5) (Bourbonnais et al. 2020; Hanson et al. 2021; Hirdes et al. 2020; Huey‐Ming et al. 2021; Puxty et al. 2019), Australia (*n* = 4) (Allen et al. 2023; Davis et al. 2019; Vilapakkam Nagarajan et al. 2022; Waird and Monaro 2021), the Netherlands (*n* = 2) (Groot Kormelinck et al. 2021; Vermunt et al. 2023) and one each from Belgium (Strauven et al. 2020), Denmark (Mortensen et al. 2022), Italy (Di Giulio et al. 2019), Sweden (Alexiou et al. 2021), Switzerland (Plüss‐Suard et al. 2020), Iran (Vedaei et al. 2023) and Singapore (Goh et al. 2021).

**TABLE 2:** Table of included studies.

First author; Date; Country	Aim of improvement	Approach to improvement	Impact
Abbott et al. 2022 USA	Implement a process for assessing and tracking delivery of care preferences	Agile methods (Krehbiel et al. 2017) used including assessment tools and technology for tracking preferences. Teams received training	Staff reported available preference information for each resident, and residents given care accordingly
Alexiou et al. 2021 Sweden	Assess sustainability of a person‐centred approach to incontinence	Staff training	The approach was considered unsustainable
Allen et al. 2023 Australia	Support staff response to early signs of resident deterioration to prevent hospital admission	Implementation of the ‘Early detection of deterioration in elderly residents guided by the Integrated‐Promoting Action on Research Implementation in Health (i‐PARIHS)’ (Harvey and Kitson 2015).	Not reported
Anaba‐Wright and Kefas 2020 UK	Prevention of pressure ulcers (PUs)	A quality council to identify how to support staff, residents and families can improve pressure ulcer care. Included teaching sessions from the community team and a resources pack	Pre‐and post‐teaching surveys showed improved confidence in identifying a developing pressure ulcer, moisture lesion and when to raise an alert.
Au et al. 2019 USA	Reduce prevalence PUs	A digital skin and wound management system consisting of a smartphone application to calculate wound length, width and surface area to allow accurate reporting and tracking	Pre‐PU prevalence (12.9%) was reduced post intervention (2.9%)
Baluyot et al. 2022 USA	Improve hospital to care home communication to prevent delays in critical medication delivery	A standardised tool for documenting medications and patient information, process underpinned by Lewin's change management theory (Lewin 1947).	Post intervention late medication administration was reduced by 52.9% for controlled medications and 77.8% for intravenous
Berning et al. 2021 USA	Conduct COVID‐19 focused advance care planning discussions	QI project, involving support from a director of palliative care and a toolkit supporting structured discussions	39% acquired a new ‘do not hospitalise’ directive. Of 52% of residents diagnosed with COVID‐19, hospitalisation rates were low (~ 2%)
Bourbonnais et al. 2020 Canada	Reduce resident distress by responding appropriately to screams	Partnership between the older person, family and formal caregivers to identify scream meanings to promote appropriate supportive action	Challenges during implementation related to caregivers' skills and organisation of work
Calderon et al. 2021 USA	Improve sepsis identification and treatment by nursing staff	Development and implementation of an educational toolkit to train nurse leaders to deliver sepsis education to care home staff.	The average score in sepsis knowledge increased from pre–post (57.5%–96.2%)
Collins et al. 2019 UK	Reduce waste and unnecessary use of oral nutritional supplements (ONS)	Plan‐Do‐Study‐Act (PDSA) (Institute for Healthcare) cycles used to understand and test strategies for reviewing, including a flow chart and collaboration.	Improved sharing of information and communication, greater consistency in prescribing and cost‐effective prescribing and deprescribing of inappropriate ONS.
Damery et al. 2021 UK	Improve the safety climate	The intervention (SPACE), informed by Learning from Excellence and Safety II approaches to QI (Mannion and Braithwaite 2017) aimed to reduce avoidable harms, accident and emergency attendance and hospital admissions through staff training in QI, support to track trends in avoidable harms and regular manager forums and celebration events	Staff perceived changes to safety climate, improvements to teamwork, working practices, information sharing, knowledge and skills. Small increases were made in scores for safety climate
Davis et al. 2019 Australia	Improve palliative and end‐of‐life care	A standardised palliative approach ‘resource kit’ including instructions for use and re‐order details for all equipment and items (e.g., soap‐free body wash, mouth emollients). QI process based on ‘facilitation model’ (Petrova et al. 2010).	Staff knowledge and confidence for discussions involving advance care planning, end‐of‐life care and supporting bereaving families increased but barriers were identified including availability of resources, lack of infrastructure, workforce issues and weak GP relationships.
Di Giulio et al. 2019 Italy	Improve end‐of‐life care for people with advanced dementia	Lecture and meetings/case discussions to discuss topics related to end‐of‐life care.	Pre, post‐intervention assessment indicated closer alignment with the recommended palliative approach including increases in feed by mouth and decreases in intravenous fluid administration and tube feeds.
Douglas and Brush 2022 USA	Improve dignity and person centredness in older people with dementia	Environmental modification (e.g., removing extra furniture, and the front of cupboards to support visualisation of resources) and improving lighting	Staff perceived reduced negative responsive behaviours and increased active engagement of people with dementia
Goh et al. 2021 Singapore	Improve incident reporting	An incident reporting system with learning elements, allowed data collation, analysis and reporting functions	Nurses reported greater awareness on patient safety issues and better communication of clinical incidents among staff
Groot Kormelinck et al. 2021 the Netherlands	Reduce inappropriate psychotropic drug use	In intervention with information and coaching implemented using the Consolidated Framework for Implementation Research (CFIR) (Damschroder et al. 2009).	The intervention evaluated positively but was time‐consuming and complex. Barriers included staff turnover, poor communication, unclear expectations and time.
Hanson et al. 2021 Canada	Decrease inappropriate antipsychotic medication use	The quality improvement intervention consisted of staff education (non‐pharmacological care), support, audit and feedback	Inappropriate antipsychotic medication use declined from 26.8% to 21.1%. Positive feedback from family, organisational leaders and staff
Harbison and Mwesige 2019 USA	Prevent inappropriate antidepressant prescribing	QI approach using education to staff, family members and residents. Residents on antidepressants were reviewed and weaned off where prescriptions were inappropriate	Antidepressants were reduced or eliminated by 25%
Hickman et al. 2020 USA	Prevent avoidable hospitalisation	Using CFIR (Damschroder et al. 2009) evaluation of an intervention to prevent avoidable hospitalisation involving staff education and direct clinical care	15/19 facilities achieved a 10% decline and 13/19 a 20% decline in hospitalisation rates
Hirdes et al. 2020 Canada	Reduce inappropriate antipsychotic use	Local QI teams given education, training and support to implement localised strategies which included mentoring, medication review strategies, networking activities and online information	The intervention was associated with increased odds that medications would be discontinued among residents without a relevant diagnosis or mental health symptoms
Hockley et al. 2019 UK	To improve palliative care	Using the PARiHS framework (Stetler et al. 2011) staff underwent a ‘train the trainer’ model to adopt the ‘PACE Steps to Success’ programme	Not reported.
Huey‐Ming et al. 2021 Canada	Prevention of falls	Implementation of Interventions for Patient Safety, a standardised, adaptable fall prevention plan displayed on patient bedside screens, posters and educational handouts	Fall rates were lower with clinical significance
Jackson et al. 2019 USA	Improve out‐of‐hours death pronouncement.	Implementation of a policy allowing registered nurses to pronounce death after hours (weekends and holidays) to avoid delays and associated family distress.	The mean time post‐intervention was less than 1/6 the average time to pronouncement preintervention.
Jenko et al. 2019 USA	Improve patient safety and satisfaction.	NAs were trained and coached on the ‘4Ps’ (potty, position, possessions and pain) to adopt in intentional rounding (IR). PDSA (Institute for Healthcare) cycles allowed modification.	Staff knowledge of IR improved, and they perceived the approach helpful and sustainable. Patient falls and lost possessions decreased
Joy et al. 2021 USA	Improve resident pain management	An intervention including pain assessment video training program. PDSAs used (Institute for Healthcare) to assess impact on nurse knowledge and resident pain	Pre and post‐test knowledge improved but no change in staff pain reporting behaviour
Kay et al. 2023 USA	Reduce hospital admissions	A paper‐based tool was used to facilitate data collection and communication between nursing staff and other providers when a resident was experiencing a change in condition. PDSA cycles (Institute for Healthcare) supported implementation and modification	Improved documentation
Lavallée et al. 2019a UK	Improve pressure injury prevention care	An intervention informed by the Conceptual Framework for Implementation Fidelity (Carroll et al. 2007) and the Theoretical Domains Framework (Cane et al. 2012)	Participants reported increased motivation and valued the bundle
Lavallée et al. 2019b UK	Improve pressure injury prevention	Used the Nominal Group technique (Van de Ven and Delbecq 1972) to gain consensus about elements to include in a care bundle, and followed steps outlined in the Behaviour Change Wheel to develop an implementation plan	Not reported
Maguire et al. 2021 USA	Pressure ulcer prevention care	An individualised turn frequency tool was implemented as a QI project. This included a decision tree, individual sensors worn by residents that transmitted ambulation status and cues to alert staff about individual repositioning needs in real‐time	Individualisation of turning schedules is feasible and safe
Mills et al. 2019 USA	Improve staff–resident interaction	A QI intervention focused on changing staff behaviour by impacting their capability, opportunity and motivation using COM‐B (Michie et al. 2014)	Evidence of interventions targeting staff behaviours being effective in improving care quality and clinical outcomes
Mortensen et al. 2022 Denmark	Improve calcium and vitamin D guidance adoption	PDSAs to implement an information sheet including the rationale for the recommendation, inclusion of need in admission meetings with new residents.	Notes audit demonstrated improvements from 32% to 65% of residents with optimal vitamin d and calcium supplements
Ouslander et al. 2021 USA	Reduce hospital admissions	Implementation of Research on Interventions to Reduce Acute Care Transfers (INTERACT), a program to reduce potentially avoidable hospital transfers, including education, champion and root cause analyses and admissions data	Barriers and facilitators to programme implementation were identified
Pimentel et al. 2020 USA	Improve frontline staff interactions with residents	COM‐B (Michie et al. 2014) used to implement a framework to assess an intervention involving identification of desired behaviours and collaboration in huddles	Intervention adaptation strategies recommended.
Plüss‐Suard et al. 2020 Switzerland	Describe antibacterial (antibiotic) use in long‐term care facilities	To optimise the use of medicines, a working group developed prescription guidelines. Quality circles, coaching, continuing education and a monitoring was implemented	Reduction in facility‐level antibacterial (antibiotic) use and invariability across care facilities
Porter et al. 2021 USA	Early identification of sepsis	A sepsis education session and screening tool implemented using Evidence‐Based Practice (ACE) Star Model (Stevens 2004).	Statistically significant improvement in staff knowledge from pre‐test to post‐test and post intervention and post‐intervention positive screens resulted in timely notification and hospital transfer
Puxty et al. 2019 Canada	Facilitate improvements in a range of outcomes including pneumonia and falls)	‘Bridges to Care’ initiative including resources, workshop and results sharing	Communication and collaboration improved
Strauven et al. 2020 Belgium	Improve medicines pathway in care homes	System Engineering Initiative for Patient Safety (SEIPS) model (Carayon et al. 2006) used to gain insight to the current pathway and support necessary change.	Identified interventions may support QI
Toles et al. 2021 USA	Improve transitions of care	The intervention included plan of care template, a toolkit, training, meetings and ongoing support. Implemented using the RE‐AIM framework (Glasgow et al. 1999) and Proctor's Framework for Implementation (Proctor et al. 2009).	Site champions reported that participating in the collaborative helped teams improve communication, quality monitoring and transitional care.
Vedaei et al. 2023 Iran	Improve oral health status of residents	Used the Joanna Briggs Institute Implementation approach (Joanna Briggs Institute 2008), delivery of educational sessions	Improved compliance with oral hygiene strategies in the follow‐up audit
Vermunt et al. 2023 The Netherlands	Support to embed QI Interventions into the routine of nursing home care	Expert coaches guided nursing homes problem analysis and improvement planning, goal setting, planning reviewed using PDSA cycles (Institute for Healthcare).	Pre compared with post‐intervention lack of adherence, 90 improved to 60%
Vilapakkam Nagarajan et al. 2022 Australia	Improve quality of palliative care provision to residents	Based on assessed barriers and facilitators, strategies to support the adoption of a palliative care model including training, evidence‐based tools and tele‐mentoring	Staff reported increased awareness, skills and confidence to discuss death and dying
Volk et al. 2020 USA	Improve daily mouth care and prevent decay gum disease	A train‐the‐trainer package delivered by hygienists to care home staff	Staff reported being able to implement the program with the coaching provided, but overall success rated by the coach and champion of each care home was moderate
Waird and Monaro 2021 Australia	Reduce incidence and severity of pressure injuries	QI project used a validated pressure injury point prevalence audit (Prentice et al. 2003) and an education package	Pressure injury prevalence reduced from 64% to 33% and suspected deep tissue injuries were eliminated post‐intervention
Wilson et al. 2019 UK	Optimise resident hydration	Strategies to improve hydration included extending drinking opportunities and extending residents' choice of fluids and a drinks menu. PDSA cycles used refine strategies	In six randomly selected residents, fluid intake increased but there was no change in the incidence of adverse health events

### 3.2 Adoption

Generic QI was the most frequently reported approach (*n* = 15) (Anaba‐Wright and Kefas 2020; Au et al. 2019; Berning et al. 2021; Davis et al. 2019; Di Giulio et al. 2019; Hanson et al. 2021; Harbison and Mwesige 2019; Hirdes et al. 2020; Huey‐Ming et al. 2021; Jackson et al. 2019; Maguire et al. 2021; Plüss‐Suard et al. 2020; Puxty et al. 2019; Strauven et al. 2020; Waird and Monaro 2021), with a smaller number of papers explicitly reporting PDSA or Plan Do Check Act (PDCA) cycles (*n* = 10) (Abbott et al. 2022; Collins et al. 2019; Damery et al. 2021; Goh et al. 2021; Jenko et al. 2019; Joy et al. 2021; Kay et al. 2023; Mortensen et al. 2022; Vermunt et al. 2023; Wilson et al. 2019). QI was combined with Lewin's change management theory (Baluyot et al. 2022) and the Consolidated Framework for Implementation Research (Ouslander et al. 2021). Two studies used ‘train‐the‐trainer’ (Calderon et al. 2021; Volk et al. 2020) and one action research (Bourbonnais et al. 2020).

Although reported as QI, several studies used approaches more readily associated with implementation. Most (*n* = 6) used the Theoretical Domains Framework, Capability, Opportunity, Motivation—Behaviour (COM‐B) or Behaviour Change Wheel (BCW) at different stages (Groot Kormelinck et al. 2021; Lavallée et al. 2019a, 2019b; Mills et al. 2019; Pimentel et al. 2020; Vilapakkam Nagarajan et al. 2022). Two used unspecified implementation approaches (Alexiou et al. 2021; Vedaei et al. 2023), and one each used the PARiHS framework (Hockley et al. 2019), the i‐PARiHS framework paired with the Medical Research Council (MRC) guide for complex interventions (Allen et al. 2023), the Consolidated Framework for Implementation Science (CFIR) (Hickman et al. 2020) and ‘intentional’ implementation (Douglas and Brush 2022). The RE‐AIM framework and Proctor's Framework for Implementation Research were used for evaluation (Toles et al. 2021). Knowledge transformation using the star model (Porter et al. 2021) was the only mention of knowledge mobilisation techniques.

Included papers reported stages of QI from inception to evaluation. Most aimed for improved clinical, or resident experience outcome, with some focusing on multiple outcomes (*n* = 39). These can broadly be categorised as optimising prescribing and medication use (*n* = 8) (Collins et al. 2019; Groot Kormelinck et al. 2021; Hanson et al. 2021; Harbison and Mwesige 2019; Hirdes et al. 2020; Mortensen et al. 2022; Plüss‐Suard et al. 2020; Strauven et al. 2020), reducing pressure injuries (*n* = 7) (Anaba‐Wright and Kefas 2020; Au et al. 2019; Damery et al. 2021; Lavallée et al. 2019a, 2019b; Maguire et al. 2021; Waird and Monaro 2021), improving assessment practices (e.g., pain, deterioration) (*n* = 5) (Allen et al. 2023; Calderon et al. 2021; Joy et al. 2021; Porter et al. 2021; Puxty et al. 2019), improving end of life care (*n* = 5) (Berning et al. 2021; Davis et al. 2019; Di Giulio et al. 2019; Hockley et al. 2019; Vilapakkam Nagarajan et al. 2022), improving resident experience (*n* = 5) (Abbott et al. 2022; Bourbonnais et al. 2020; Douglas and Brush 2022; Mills et al. 2019; Pimentel et al. 2020), reducing unnecessary hospital admissions (*n* = 5) (Damery et al. 2021; Hickman et al. 2020; Kay et al. 2023; Ouslander et al. 2021; Toles et al. 2021), reducing falls (*n* = 4) (Damery et al. 2021; Huey‐Ming et al. 2021; Jenko et al. 2019; Puxty et al. 2019), improving oral care (*n* = 2) (Vedaei et al. 2023; Volk et al. 2020), optimising hydration (*n* = 1) (Wilson et al. 2019) and improving continence care (*n* = 1) (Alexiou et al. 2021). Three studies focused on specific staff activities including, timely death pronouncement (Jackson et al. 2019), communication with hospital staff (Baluyot et al. 2022) and incident reporting (Goh et al. 2021). One focused on the process of embedding complex QI interventions into routine nursing home care (Vermunt et al. 2023).

### 3.3 Impact

The stage of improvement reported in papers included (i) assessment of current practice or determinants of care (*n* = 4), (ii) intervention design or adaptation (*n* = 3), (iii) adoption of improvement interventions (*n* = 29) and (iv) evaluation (*n* = 8). Those using PDSA cycles referred to several stages, but for brevity these have been included in the ‘adoption of improvement stage’ reported below.

*Assessment of current practice* was based on qualitative interviews and observation (Strauven et al. 2020) and documentary review (Collins et al. 2019), assessment of determinants was theoretically underpinned using action research methods (Bourbonnais et al. 2020) and COM‐B‐based interviews (Mills et al. 2019). *Design and adaptation* included two adaptations of existing interventions to local context (Allen et al. 2023; Hockley et al. 2019) and development of a care bundle for implementation in nursing homes (Lavallée et al. 2019b). *Adoption* studies generally reported quantitative outcomes of varying impact, generally short term and not generalisable due to small sample numbers. Outcomes included a reduction in delayed medication administration (Baluyot et al. 2022), a modest decline in inappropriate antipsychotic use (Hanson et al. 2021; Hirdes et al. 2020), reduction or elimination of antidepressants (Harbison and Mwesige 2019) an increase in vitamin d and calcium supplements (Mortensen et al. 2022), reduction in antibacterial (antibiotic) use (Plüss‐Suard et al. 2020), reduced adverse events (e.g., pressure ulcer development) (Anaba‐Wright and Kefas 2020; Au et al. 2019; Lavallée et al. 2019a; Maguire et al. 2021), falls (Jenko et al. 2019), negative behaviours (Douglas and Brush 2022) and increased resident safety (Vermunt et al. 2023). There were varied results in attempts to decrease unnecessary hospitalisations with some studies reporting increased rates of ‘do not hospitalise directives’ (Berning et al. 2021) or measurable reductions in admissions (Hickman et al. 2020) and others did not report measurable improvements (Kay et al. 2023; Toles et al. 2021). In relation to oral hydration and nutrition (Vedaei et al. 2023; Volk et al. 2020; Wilson et al. 2019), tangible outcomes were not reported, but there was positive feedback from staff. Recognition of deterioration in health interventions led to better staff knowledge (Calderon et al. 2021; Porter et al. 2021) and in one case, timely escalation of care (Porter et al. 2021). Post intervention, staff reported interventions improved collaboration (Puxty et al. 2019) and knowledge about pressure ulcer prevention (Anaba‐Wright and Kefas 2020) or palliative and end of life care (Davis et al. 2019). Three implementation studies reported interventions had no impact on quality or outcome (Alexiou et al. 2021; Joy et al. 2021; Ouslander et al. 2021). In one case anecdote was used to imply improvement (Abbott et al. 2022). *Evaluation* took the form of identifying need for or devising adaptations to interventions (Groot Kormelinck et al. 2021; Pimentel et al. 2020; Vilapakkam Nagarajan et al. 2022), measuring falls and identifying staff ‘buy‐in’ as a component of successful change (Huey‐Ming et al. 2021; Waird and Monaro 2021), qualitative interviews (Goh et al. 2021) and quantitative measures indicating improvements in resident safety and comfort (Damery et al. 2021; Di Giulio et al. 2019).

## 4 Discussion

The aim of our review was to understand how QI strategies had been adopted and how impact had been assessed in care homes for older people. In total, 44 papers met the inclusion criteria. Most studies took place in the United States (*n* = 19), used a generic QI approach (*n* = 15) and the most common areas of focus were prescribing (*n* = 8), pressure ulcer prevention (*n* = 6), resident assessment (*n* = 5) and falls reduction (*n* = 4). Outcomes were largely quantitative, of varying impact and generally short term.

This narrative review (*n* = 44 included papers) updates a previous scoping review of studies from 2000 to 2019 (*n* = 75 included papers) (Chadborn et al. 2021). A total of 119 papers across a total time span of over two decades (2000–2024) demonstrates the limited attention given to improving quality in care homes, although, there is a slow but steady increase in publications. Given the number of care home residents and the complexity of their care needs, and by comparison with the vast number of publications about quality improvement in primary and secondary care, this is a significant under representation. The range of improvements focused on few practices, generally those externally measured, for example, falls, pressure ulcer prevention and medicines management. Other care activities are important to residents, but they were rarely engaged in prioritising or subsequent QI activity. Although our focus was not on who was engaged in supporting improvement, we found no explicit examples of resident inclusion. We consider this a serious omission. Individual resident priorities differ. A published review identified commonalities in their care quality indicators, including staff behaviours, continuity and routine, ability to make choices, dignity and culture (Gilbert et al. 2021), areas that very few of our included papers considered. Staff engagement was also variable and their ‘buy‐in’ was hard to assess. In some instances, external teams appeared to be the driving force for improvement and little reference was made to either staff involvement or post‐improvement sustainability.

Although there were potential opportunities for shared learning, these were generally not realised. There was little reference to sharing best practice or learning from existing initiatives, either from the literature or from other care homes. Impact was variable, but in most studies, changes were poorly reported or were modest. Replication of strategies or interventions, in many cases, would not be possible due to limited details reported. Multiple interventions for the same problem suggest duplication of effort. Poor reporting is a common problem in QI (Jones et al. 2019).

Our intention was to build on a previous review (Chadborn et al. 2021), therefore our search focused on quality improvement papers. Data extraction revealed some reference to implementation and knowledge mobilisation. These were evident in our included papers, through direct reference to implementation (*n* = 13), knowledge mobilisation (*n* = 1) or associated strategies or frameworks. Both approaches are frequently used in primary and secondary care. Therefore, for completeness, we reviewed the wider literature using the terms ‘implementation’ and ‘knowledge mobilisation’ alongside ‘care homes’ and found no additional studies published in our timeframe (2019–2024).

The predominant use of QI strategies in care homes limits options. Evidence of QI effectiveness remains “very mixed”, because QI is: (i) applied to short term, small‐scale projects, (ii) led by practitioners with limited QI knowledge or influence to make change, (iii) strategies not adapted to context, (iv) limited evaluation and (v) a lack of sharing to ‘develop collective solutions’ (Dixon‐Woods et al. 2012). These problems are likely to be amplified in care homes as QI is relatively underdeveloped in this context and the singular nature of care homes. Experts in QI suggest two immediate challenges: convincing people there is (i) a problem and (ii) an achievable and impactful solution (Dixon‐Woods et al. 2012). As many QI initiatives in our review were driven and delivered by external sources, these challenges were poorly considered, thus limiting the potential to manage downstream issues.

Although, the problems of QI in health care generally (Dixon‐Woods and Martin 2016), and in care homes specifically (Masso et al. 2014) are well documented, concrete recommendations for delivering QI in care homes are absent. From our review we offer the following tangible recommendations for the adoption of evidence‐based practice in care homes. Importantly, care homes should not be limited to QI alone, the most appropriate evidence to practice strategy should be selected, be it improvement, implementation or knowledge mobilisation. There are a wealth of theories, models and frameworks to support initiatives designed to improve healthcare outcomes and experiences, all have potential but can be impenetrable particularly for staff who are not familiar with them. Care home staff know their setting and their residents best and therefore should be the main actors in improvement efforts. Engaging staff and residents has the potential to improve aspects of care that are important in *that particular* home and improve staff morale and resident experience. Based on our synthesis of available literature and identification of current limitations to improving resident experiences and outcomes we offer the following principles as a basis to guide any initiative to improve resident experiences and outcomes.

1. *Establish the ‘project’ team*. Bring together front‐line staff, residents and relevant experts (e.g., clinicians, managers, ‘improvers’).
2. *Move away from a deficit model*. Consider what is done well, how this is achieved and how this may inform other areas.
3. *Prioritise areas for improvement*. In addition to clinical and externally driven areas, consider also resident priorities and their definition of quality care.
4. *Identify ‘best practice’*. Review evidence and guidelines and work with experts to adapt these to local context. Most guidelines are written for primary and secondary care and may not easily translate to the care home context. Evidence‐based (best) care melds research evidence, practitioner expertise and resident preference (Straus et al. 2018).
5. *Decide how to measure improvement*. Establish the data to be collected, for example, quantitative (e.g., numbers and severity of pressure ulcers, falls) and qualitative (e.g., resident and staff observation and opinion) and how and when comparisons will be made (Donabedian 1988).
6. *Understand the challenges to best practice*. Talk to staff, residents and visitors and observe care.
7. *Co‐design strategies to effect change*. Co‐designed strategies are likeliest to be effective (Greenhalgh et al. 2016). Strategies should directly address challenges (Baker et al. 2015) and not rely on the ‘norm’ of training. Some strategies work in some contexts with some people but there are no ‘magic bullets’ (Eccles and Grimshaw 2004).
8. *Sustain the improvement*. Consider ‘light touch’ ongoing (but diminishing) monitoring and communication, access to any materials, maintaining staff motivation and recognition (Devi et al. 2023).
9. *Share good practice*. Share achievements and learn about what strategies worked for others.
10. *Clear, detailed and accessible reporting*. Report successes in a way that allows others to replicate (Hoffmann et al. 2014).

## 5 Conclusion

The literature demonstrates a steady upward trajectory of quality improvement in care homes. There appears to be a recent tentative move towards use of other theories, models and frameworks with strategies including implementation and knowledge mobilisation. Reporting is generally limited, which prevents replication of improvement strategies. The focus of improvement is generally on clinical, externally driven areas rather than resident priorities. We advocate application of the 10 straightforward principles listed above to support optimum care home practice.

## Author Contributions

F.C. conceived the idea for the review; methods were designed by all three authors. Data were extracted by all three authors and H.H. curated the data. H.H. drafted the manuscript, and all named authors revised and approved the final version. All authors agree to be personally accountable for the accuracy and integrity of the work.

## Ethics Stateme1nt

The authors have nothing to report.

## Conflicts of Interest

The authors declare no conflicts of interest.

## Supporting information


Data S1.

Data S2.

Data S3.

## Data Availability

Data sharing not applicable to this article as no datasets were generated or analysed during the current study.
