# Supplementary material for: How Have Quality Improvement Strategies Been Adopted and How Has Impact Been Assessed in Care Homes for Older People? A Systematic Search and Narrative Review
Source: Int J Older People Nurs. 2025 Sep 5;20(5):e70036. doi: 10.1111/opn.70036 (PMC12412655; doi:10.1111/opn.70036)
Supplement: Supplementary file 2 — Data S2. [file OPN-20-e70036-s001.docx]

**Supplementary file2: Full search strategy**

***Database - CINAHL Plus with Full Text***Interface - EBSCOhost Research Databases
Search Screen - Advanced Search

| S1 | "ltcf" OR "institutional care" OR “snf" OR "residential home" OR (MH "skilled nursing facilities") OR "care home" OR (MH "institutionalization") OR (MH "residential facilities") OR (MH "long-term care") OR (MH "homes for the aged") OR (MH "nursing homes") | **Search modes** - Boolean/Phrase | 91976 |
| --- | --- | --- | --- |
| S2 | (MH "quality improvement") OR (MH "total quality management") OR “pdsa” OR “process improvement” OR (MH "health services research") OR (MH "quality indicators, health care") OR "model for improvement" OR “six sigma” | **Search modes** - Boolean/Phrase | 99769 |
| S3 | S1 AND S2 | **Search modes** - Boolean/Phrase | 2842 |
| S4 | S1 AND S2 | **Limiters** - Published Date: 20190101-20241231  **Search modes** - Boolean/Phrase | 301 |
| S5 | S1 AND S2 | **Limiters** - Published Date: 20190101-20241231  Narrow by Language: - english  **Search modes** - Boolean/Phrase | 295 |

***Database – MEDLINE****Interface - EBSCOhost Research Databases
Search Screen - Advanced Search*

| S1 | (MH "skilled nursing facilities") OR (MH "institutionalization") OR (MH "residential facilities") OR (MH "residential care") OR (MH "long term care") OR (MH "nursing homes") OR (MH "homes for the aged") OR (MH "residential facilities") | **Search modes** - Boolean/Phrase | 65009 |
| --- | --- | --- | --- |
| S2 | (MH "quality improvement+”) OR (MH "quality indicators, health care”) OR (MH "health services research") OR (MH "total quality management") OR “Six Sigma” OR “pdsa” | **Search modes** - Boolean/Phrase | 96055 |
| S3 | S1 AND S2 | **Search modes** - Boolean/Phrase | 2977 |
| S4 | S1 AND S2 | **Limiters** - Published Date: 20190101-20241231  **Search modes** - Boolean/Phrase | 696 |
| S5 | S1 AND S2 | **Limiters** - Published Date: 20190101-20241231  Narrow by Language: - english  **Search modes** - Boolean/Phrase | 677 |

***Database – APA PsycINFO****Interface - EBSCOhost Research Databases
Search Screen - Advanced Search*

| S1 | "ltcf" OR "institutional care" OR "snf" OR "residential home" OR "skilled nursing facilit*" OR "care home*" OR "residential facilit*" OR "long term care" OR "homes for the aged" OR "nursing home*" OR "residential care" | **Search modes** - Boolean/Phrase | 26754 |
| --- | --- | --- | --- |
| S2 | "quality improvement" OR "total quality management" OR “pdsa” OR “process improvement” OR "health services research" OR "model for improvement" OR “six sigma” OR "quality indicator” | **Search modes** - Boolean/Phrase | 29513 |
| S3 | S1 AND S2 | **Search modes** - Boolean/Phrase | 1539 |
| S4 | S1 AND S2 | **Limiters** - Published Date: 20190101-20241231  **Search modes** - Boolean/Phrase | 275 |
| S5 | S1 AND S2 | **Limiters** - Published Date: 20190101-20241231  Narrow by Language: - english  **Search modes** - Boolean/Phrase | 268 |

***Database - ASSIA****Interface - ProQuest
Search Screen - Advanced Search*

"ltcf" OR "institutional care" OR “snf" OR "residential home*" OR "skilled nursing facilit*” OR "care home*" OR "institutionali$ation" OR "residential facilit*" OR "long term care" OR "homes for the aged" OR "nursing home*"

AND

"quality improvement" OR "total quality management" OR “pdsa” OR “process improvement” OR "health services research" OR "quality indicators, health care" OR "model for improvement" OR “six sigma”
