# Supplementary material for: How Have Quality Improvement Strategies Been Adopted and How Has Impact Been Assessed in Care Homes for Older People? A Systematic Search and Narrative Review
Source: Int J Older People Nurs. 2025 Sep 5;20(5):e70036. doi: 10.1111/opn.70036 (PMC12412655; doi:10.1111/opn.70036)
Supplement: Supplementary file 3 — Data S3. [file OPN-20-e70036-s003.docx]

**Supplementary file 3: Data extraction template**

| **First author**  **Date**  **Country** | **Aim of improvement** | **Approach to improvement** | **Impact** |
| --- | --- | --- | --- |
|  |  |  |  |
|  |  |  |  |
|  |  |  |  |
|  |  |  |  |
|  |  |  |  |
